# Medical Problems

**Published:** 1847-06

**Authors:** William Griffin

**Affiliations:** Physician to the County of Limerick Infirmary


*Medical Problems.* By WILLIAM GRIFFIN, M. D., Physician to the County of Limerick Infirmary.

*When miscarriage or premature labour takes place at fixed periods, from the influence of acquired habit, may not the periodical movements be prevented by such remedies as prevent the recurrence of an epileptic fit or a paroxysm of ague?*

I was called on some years since to attend Mrs. C., a lady who was ill with the usual symptoms of miscarriage at the third month. She informed me, that she had had a miscarriage at the end of the third month of her first pregnancy. She reached nearly to her full time on the second occasion, fell into puerperal convulsions in her labour, and was delivered of a dead child. In her next pregnancy she had a miscarriage at three months; in her fourth at three months; and now in her fifth she was again threatened exactly at the same period. She informed me that everything had been done to prevent it. She had been bled repeatedly, kept for weeks upon low diet, and was confined during the time entirely to the horizontal position. She lived, in fact, between the bed and the sofa. In this new attack some friends recommended her to send for me, with the hope of having some plan of treatment devised by which she might be enabled to go on to her full time. The amount of the hæmorrhage was, however, so considerable, and the uterine pains so general and regular, I told her it was impossible to prevent the miscarriage, but if I was informed of her condition on any future occasion, when six weeks or two months should elapse, I might, perhaps, succeed. Miscarriage, I believe, took place on that night or on the next morning.

In three or four months afterwards I received an intimation from this lady that she was two months pregnant. On considering the probable causes of the previous miscarriages, I could not detect any very obvious one. Her health was excellent, her habits regular, her diet moderate. The extreme regularity with which the miscarriage always occurred at the end of the twelfth week rather confirmed the only conjecture I could form, that it depended wholly on the influence of an acquired habit; and the question necessarily arose, how was this acquired habit to be interrupted or controlled? All the ordinary measures had already been adopted, and the poor lady had been subjected for weeks to the most irksome and tantalizing restrictions, without the slightest advantage. In this difficulty it occurred to me, that as periodical attacks of epilepsy may often be prevented by a long course of any of the metallic tonics, the periodical movements connected with the action of the uterus might be also under their control. I therefore directed my patient to take two and a half grains of oxide of zinc, with two grains of extract of hops, three times a day, and after each pill, two table-spoonsful of a mixture of valerian, aromatic spirits of ammonia, and infusion of snake root. She was also ordered a box of pills, containing a grain of opium in each, one of which she was to take when pain came on, and to repeat the dose every hour until relief was obtained. As she was of a nervous habit, I thought, if my view of the case was a correct one, that both bloodletting and confinement to the sofa would rather tend to increase than lesson the danger, by weakening the general tone of the system, and rendering her more susceptible of slight impressions. I therefore advised her, instead of lying all day upon the sofa, to keep out in the open air on fine days as much as possible, without, however, fatiguing herself, and to live in the manner she usually found to agree best with her. Under this plan of treatment, she passed the twelfth week without the slightest threatening, to her very great joy and the gratification of her friends. Happening, however, in about a fortnight afterwards, to visit a sister who was very ill, she was so shocked at her appearance that she was immediately seized with the usual symptoms premonitory of miscarriage. She had a discoloured leucorrhœal discharge, which, in a few hours, was followed by uterine pains, being exactly the symptoms which had ushered in all her former attacks. She took the opium pills as I had directed her, and before morning the pains and discharge had all subsided, and in a day or two she was as well as she had been before. She then resumed the zinc and valerian for three or four weeks, after which period I did not consider it necessary to continue them. She went on to her full time without the slightest uneasiness, and was finally delivered of a fine child, which is now well and thriving.

Very soon after this lady had applied to me, and when I had just obtained strong presumptive evidence of the success of the treatment adopted, Mrs. H. consulted me with a view of obtaining advice as to the best means of preventing premature labour, which, she feared, was about to come on. It had already occurred to her four times successively; the infant dying in the middle of the sixth month, and her delivery of a dead child taking place at the end of it. She had now completed the fourth month of her pregnancy. On making some inquiries to ascertain whether she had had at any time a syphilitic affection, I could only glean, that she had suffered with soreness in the vagina for three or four months after her marriage, for which mercurials had been prescribed. This was obviously a very different case from the one already related. In the latter, hæmorrhage and pain came on first, and the child died as a consequence. In the former, the child died in the first instance, and premature labour followed. In Mrs. C.'s case the mere influence of habit, the tendency in the constitution to be influenced periodically, brought on labour. In Mrs. H.'s case the infant died through some unknown cause, and labour came on because of its death. There did not appear, therefore, to be any analogy which could suggest a treatment precisely similar. Taking into consideration the probability of the child's death being occasioned by some syphilitic taint in the habit, I therefore decided on giving calomel and opium in small doses, so as to affect the gums slightly; and subsequently with a view of preventing the accession of labour at the end of the sixth month, from the influence of habit, to adopt the same plan which had been pursued so successfully in the case of Mrs. C. After a fortnight or three weeks the gums became sore, upon which the calomel was suspended, and pills of oxide of zinc, with the valerian mixture prescribed for Mrs. C., were substituted. Under this treatment, Mrs. H. passed the usual period at which labour came on, and continued in good health to the 7th July, when she was attacked with griping pains and slight flooding. These symptoms subsided by keeping perfectly at rest, and taking a few anodyne pills. On the 17th of the same month, when she had reached within four weeks of her full time, she was seized with threatenings of labour, and on the 19th was delivered of a living child, which died after some hours. This lady resided in the country, at a considerable distance from me, and could not receive that immediate attention and advice, which, if she had been in town, would probably have enabled her to go to her full time.

About the same time these cases were under my care, I was consulted by Mrs. A., who had also been seized with premature labour, in consequence of the infant dying in the seventh month for three successive years. In her last labour she was seized with violent puerperal convulsions, during which she was delivered of an infant, which had evidently been dead for many days.

I had not had the medical management in the earlier labours, and was merely called in a little before the lady's confinement; and in the last I had, therefore, no opportunity of adopting any preventive treatment. When she was again pregnant, however, and approached the seventh month, I adopted the same treatment as I had done in the former cases, partly to counteract, if possible, any tendency to labour arising from acquired habit, and partly that I thought it not impossible the same influence which was capable of controlling a periodical movement in the system comprehending months, might also control causes tending to the death of the child. The lady took the oxide of zinc pills and valerian mixture, three times a day, for some weeks before the period when labour might be expected; and she had opium pills by her, one of which she was directed to take whenever she was seized with uterine pains. These last she had no occasion to take, having gone on remarkably well to her full time, when she fell into natural labour, and was delivered of a living child: it expired, however, almost immediately after. It was obvious here, that the treatment had actually accomplished both the objects I had in view; it had broken up the morbid habit, and it had so interfered with the poisonous influence which had heretofore so invariably, in the seventh month, caused the death of the child, that the latter was born alive. Its death so soon after birth, without any obvious cause, suggested the possibility of some syphilitic taint in the parents, which led to very particular inquiries. The father, it appeared, had not had a syphilitic affection for ten years before his marriage, and never had one since. Acting, however, on the possibility that, even after that long period, some deleterious influence might have been communicated to the mother, and thus evinced itself in the feeble vitality of the offspring, I placed the lady, as soon as she was out of her confinement, under a mild course of calomel (one grain every night, until her gums became tender,) and again, when she reached the dangerous period, resorted to the zinc and valerian. I had now the happiness of finding all my hopes realized; she went to her full time, and had a fine living infant, which has since been going on well.

In the first of the cases I have given, in which abortion occurred apparently from the acquired habit, the treatment was quite successful. The disposition to premature action in the womb was controlled exactly as the movements to a fit of epilepsy or of ague might have been arrested by some similar means. Quinine, carbonate of iron, or nitrate of silver, might have accomplished the object probably as well as the oxide of zinc and valerian. The latter were preferred chiefly because I believed they would be less likely to injure the fœtus, but also because I had considerable confidence in the influence which both, and especially which large doses of valerian, possess over the nervous movements. In the second case, the lady, who had fallen into labour on four successive occasions at the sixth month, in consequence of the death of the child, carried her child to the eighth month, and it was born alive. This instance, however, can hardly be adduced as evidence of the influence of the zinc and valerian, as it seems probable the death of the child, and consequent premature labour, were owing to some syphilitic taint, which was removed by the mercurial treatment. In the third case,—that of Mrs. A. Z.,—the inference as to the truth of the principle assumed may be considered more satisfactory, as she reached her full time, and had even a living child before the mercurial treatment was adopted.

These cases are so few in number that I offer them to the profession as evidence of the novel application of a principle long recognised in the treatment of epilepsy, ague, and other periodical diseases, with some diffidence. The legitimate manner, however, in which the analogy was inferred, and the remarkable success attending the remedial measures is suggested, were too striking not to make a deep impression on my own mind.

The extreme difficulty, too, which practitioners so often feel in the prevention of abortion and premature labour, as well as the deep interest which married people naturally attach to successful treatment in such cases, invest suggestions supported by even a very limited experience with some importance. The valerianate of zinc, which was not in use at the time these cases were under treatment, would have been a far more desirable preparation, and probably quite as effective. Where it is necessary to continue medicines of this class for a long period, it is a great object to be in possession of such an elegant substitute for so disagreeable a mixture as the valerian.—*Dublin Quart. Journ. of Med. Sci.*

